# CVB3 Inhibits NLRP3 Inflammasome Activation by Suppressing NF-κB Pathway and ROS Production in LPS-Induced Macrophages

**DOI:** 10.3390/v15051078

**Published:** 2023-04-28

**Authors:** Yanqi Wang, Zhirong Sun, Hongkai Zhang, Yahui Song, Yi Wang, Wei Xu, Min Li

**Affiliations:** 1 Institute of Biology and Medical Sciences, Soochow University, Building 703, 199 Ren-ai Road, Suzhou 215123, China; wangyanqi96@163.com (Y.W.); sunzhirong97@126.com (Z.S.); syh20174252019@126.com (Y.S.); m15172876361@163.com (Y.W.); 2 Suzhou Center for Disease Prevention and Control, 72 Sanxiang Road, Suzhou 215004, China; 20184252007@stu.suda.edu.cn

**Keywords:** NLRP3 inflammasome, coxsackievirus B3, macrophages

## Abstract

Inflammasomes are cytosolic sensors of pathogens. Their activation can lead to the induction of caspase-1-mediated inflammatory responses and the release of several proinflammatory cytokines, including IL-1β. There is a complex relationship between viral infection and the nucleotide-binding oligomerization domain-like receptors family pyrin domain-containing 3 (NLRP3) inflammasome. The activation of the NLRP3 inflammasome is essential for antiviral immunity, while excessive NLRP3 inflammasome activation may lead to excessive inflammation and pathological damage. Meanwhile, viruses have evolved strategies to suppress the activation of inflammasome signaling pathways, thus escaping immune responses. In this study, we investigated the inhibitory effect of coxsackievirus B3 (CVB3), a positive single-strand RNA virus, on the activation of the NLRP3 inflammasome in macrophages. CVB3-infected mice had significantly lower production of IL-1β and a lower level of NLRP3 in the small intestine after LPS stimulation. Furthermore, we found that CVB3 infection inhibited NLRP3 inflammasome activation and IL-1β production in macrophages by suppressing the NF-κB signaling pathway and ROS production. Additionally, CVB3 infection increased the susceptibility of mice to *Escherichia coli* infection by decreasing IL-1β production. Collectively, our study revealed a novel mechanism of NLRP3 inflammasome activation by suppressing the NF-κB pathway and ROS production in LPS-induced macrophages. Our findings may provide new ideas for antiviral treatment and drug development for CVB3 infection.

## 1. Introduction

Coxsackievirus B3 (CVB3), a positive single-strand RNA virus, belongs to the enterovirus B genus of the Picornaviridae family. CVB3 is recognized as a major etiological factor of viral myocarditis (VMC) [1]. VMC is a serious condition that may progress to dilated cardiomyopathy and even heart failure, and accounts for nearly 50% of the indications for heart transplantation [2]. Currently, there is accumulating evidence that the progression of VMC is caused by the direct virus-induced damage of cardiomyocytes in the early stage, and indirect inflammatory injury induced by virus-activated immune responses in the late stage. In the long term, uncontrolled inflammation may play a decisive role in the progression of VMC [1,3]. Therefore, it is of great importance to further elucidate the interaction between CVB3 infection and host immune responses, which will uncover the pathogenesis of VMC and facilitate the development of potential therapeutic targets.

The innate immune response is the first line of defense against viral infections. Viruses can be recognized by pattern-recognition receptors, such as Toll-like receptors (TLRs), retinoic acid-inducible gene I-like receptors (RLRs), and nucleotide-binding oligomerization domain (NOD)-like receptors (NLRs), triggering the innate immune response [4]. Many studies have been conducted on the role of TLRs and RLRs in VMC pathogenesis [5]. However, the interaction between CVB3 and NLRs is less studied. Among the NLR inflammasome complexes, the NLR family pyrin domain-containing 3 (NLRP3) inflammasome is one of the most widely studied and is a crucial regulator in the maturation of two pro-inflammatory interleukin (IL)-1 family cytokines, IL-1β and IL-18 [6]. NLRP3 inflammasome activation leads to the recruitment of the apoptosis-associated speck-like protein containing a C-terminal caspase recruitment domain (ASC), and results in the activation of pro-caspase-1 and the formation of active caspase-1. Caspase-1 is known as an IL-1-converting enzyme and can cleave the pre-forms of IL-1β and IL-18 into their active forms. The activation of the NLRP3 inflammasome usually requires two signals. The first signal is the priming signal induced by TLR, which then activates nuclear factor (NF)-κB-dependent proinflammatory cytokine transcription. The second signal is delivered from potassium efflux, calcium influx, reactive oxygen species (ROS), or lysosomal rupture, and can trigger the assembly of a multi-protein complex consisting of NLRP3, the adaptor protein ASC, and pro-caspase-1 [7].

Inflammasome activation is involved in host immune responses to a wide range of microbial pathogens and endogenous danger signals through the regulation of caspase-1-induced inflammatory responses. Meanwhile, many pathogens have evolved strategies to suppress the activation of inflammasome signaling pathways and escape immune responses. An increasing number of studies have reported a relationship between viral infection and NLRP3 inflammasome activation [5]. CVB3 infection can directly activate the NLRP3 signaling pathway in cardiomyocytes [8], while CVB3 viral proteins can also directly degrade NLRP3, thus inhibiting its activation [9,10]. In 2017, Xin et al. reported that HBV infection inhibited NLRP3 inflammasome activation in lipopolysaccharide (LPS)-activated macrophages [11]. Macrophages play an important role in host immunity against infection. However, the regulatory effect of CVB3 on the NLRP3 signaling pathway in macrophages is unknown. We addressed this issue by using CVB3-infected mice and macrophages. We found that CVB3 inhibited NLRP3 inflammasome activation by suppressing the NF-κB pathway and ROS production in LPS-induced macrophages. Our findings may provide information on the novel inhibitory mechanism of CVB3 infection in the innate immune response.

## 2. Materials and Methods

### 2.1. Virus and Cells

CVB3 (Nancy strain) was kindly provided by Professor Yingzhen Yang (Key Laboratory of Viral Heart Diseases, Zhongshan Hospital, Shanghai Medical College of Fudan University, Shanghai, China), and was serially passaged in HeLa cells.

Murine peritoneal macrophages were collected from C57BL/6 mice on day 4 post-intraperitoneally injection with thioglycollate medium (2 mL, 4% per mouse), and cultured in RPMI 1640 supplemented with 10% FBS (Gibco, Carlsbad, CA, USA), 100 units/mL penicillin, and streptomycin at 37 °C in the presence of 5% CO_2_.

### 2.2. Mice and Treatments

The animal experiments were performed in accordance with Soochow University institutional guidelines, and the study was approved by the Ethics Committee of Soochow University in written form (201909A350). The euthanasia of mice was performed via carbon dioxide inhalation with minimum fear, anxiety, and pain. Male BALB/c mice aged 6–8 weeks old were purchased from Shanghai Slac Animal Center and housed in an SPF facility.

Mice were infected intraperitoneally with 200 µL PBS containing a 1000 PFU dose of CVB3. The small intestines were collected at the indicated time. In the *Escherichia coli* infection experiment, mice were infected intraperitoneally with 2.0 × 10^7^ CFU of *E. coli* (ATCC25922) 3 days post-infection with CVB3. After 18 h, peritoneal lavage was conducted for analyzing the bacterial load and inflammatory cytokine levels. Individual experiments were conducted at least three times with 6 to 8 mice per group.

### 2.3. Western Blotting

Cells or grinding tissue were lysed in RIPA buffer containing a protease and phosphatase inhibitor cocktail (510026, 410043, Bimake, Shanghai, China). The protein concentrations were detected using a BCA protein quantification kit (K3000, Biocolor, Carrickfergus, UK). Then, the same amounts of protein were separated using SDS-PAGE and transferred onto PVDF membranes (IPFL00010, Merck, Rahway, NJ, USA). Sodium dodecyl sulfate polyacrylamide gel electrophoresis (SDS-PAGE) and Western blot analysis were performed with 40 mg protein. The cell lysates were detected with the following antibodies: anti-NLRP3 (E71610-93, Huaan Biotechnology, Jinan, China), anti-caspase-1 (E71608-69, Huaan Biotechnology, Jinan, China), anti-IL-1β (A1112, ABclonal, Shenzhen, China), anti-GADPH (FD0063, Fude Biotechnology, Hangzhou, China), anti-β-actin (A5441, Merck, Rahway, NJ, USA), anti-p47-phox (sc-17845, Santa Cruz Biotechnology, Dallas, TX, USA) and anti- Na^+^/K^+^ATPase (sc-21713, Santa Cruz Biotechnology, Dallas, TX, USA), ASC (A1170, ABclonal, Shenzhen, China), anti-phospho-p65 (3033S, CST, Boston, MA, USA), anti-p65 (8242S, CST, Boston, MA, USA), IKK-β (8943S, CST, Boston, MA, USA), and Phospho-IKK-β (2594S, CST, Boston, MA, USA). This was followed by incubation with a horseradish peroxidase-conjugated secondary antibody (4030-05, 1034-05, Southern Biotechnology, Birmingham, AL, USA).

### 2.4. Reverse Transcription and Real-Time PCR Analysis

The extraction of total RNA was performed using a RNAiso reagent (No. 9109, Takara, Shiga, Japan), and cDNA was synthesized via reverse transcription (No. DRR063A, Takara, Shiga, Japan). Quantitative real-time RT-PCR (RT-PCR) was performed using SYBR green real-time PCR kits (No. DRR041A, Takara, Shiga, Japan) on an Eppendorf Realplex using the following primers (Table 1).

### 2.5. Measurement of ROS Production

Murine peritoneal macrophages were seeded at a density of 1.0 × 10^5^ per well in 96-plate well, and then, stimulated with CVB3 at MOI = 5. After 24 h, the cells were treated with LPS (1 μg/mL) for 1 h of incubation. After washing three times with PBS, for the detection of mitochondrial ROS, the cells were incubated with 10 μM DCFH-DA (Solarbio, Beijing, China, D6470) for 20 min at 37 °C, and then, washed three times with PBS. Fluorescence was monitored using a fluorescence microplate reader with excitation at 488 nm and emission at 525 nm. The results are presented as the fold change relative to the control group.

### 2.6. Enzyme-Linked Immunosorbent Assay (ELISA)

The release of IL-6, IL-1β, or TNF-α in cell supernatants or in the peritoneal lavage fluid was analyzed using ELISA assay kits according to the manufacturer’s instructions (eBioscience, San Diego, CA, USA).

### 2.7. Histologic Examination and Pathological Score

The mice were sacrificed, and then, their small intestines or lungs were excised, fixed with 10% neutral phosphate-buffered formalin, and then, embedded in paraffin. Sections 5 μm thick were stained with hematoxylin and eosin for light-microscopic examination, to assess small intestine and lung injury and inflammation. The sections were graded by an experienced pathologist who was unaware of the specimens’ status for damage to the lung and small intestine, according to the relative lesion area, necrosis, and inflammatory infiltration. Lung and small intestine injury were scored according to the following criteria [12]: (1) alveolar congestion, (2) hemorrhage, (3) the infiltration or aggregation of neutrophils in the airspace or vessel wall, and (4) the thickness of the alveolar wall. For each subject, a five-point scale was applied: 0: minimal (little) damage; 1+: mild damage; 2+: moderate damage; 3+: severe damage; and 4+: maximal damage. The points were added up and are expressed as the median score ± range of injury. Small intestinal mucosal injuries were graded using Chiu’s method [13]: 0: normal small intestinal mucosal villi; 1: the development of subepithelial Gruenhagen’s space, usually at the apex of the villus and often with capillary congestion; 2: extension of the subepithelial space with moderate lifting of epithelial layer from the lamina propria; 3: massive epithelial lifting down the sides of the villi (a few tips may be denuded); 4: denuded villi with lamina propria and dilated capillaries exposed (increased cellularity of the lamina propria may be noted); and 5: digestion and disintegration of the lamina propria, as well as hemorrhage and ulceration.

### 2.8. Statistical Analysis

GraphPad Prism 8.0.1 Software (GraphPad Software, La Jolla, CA, USA) was used to perform the statistical analysis. A two-tailed Student’s test, ANOVA with Tukey’s multiple comparisons post-test, and Pearson’s correlation analysis were performed for statistical comparisons. All statistics analysis data are expressed as mean ± standard error of the mean. All *p* values were two-sided, and a *p* value < 0.05 was considered statistically significant. All experiments were performed at least three times.

## 3. Results

### 3.1. CVB3 Inhibits NLRP3 Expression and IL-1β Production in the Small Intestine

**Figure 1:**
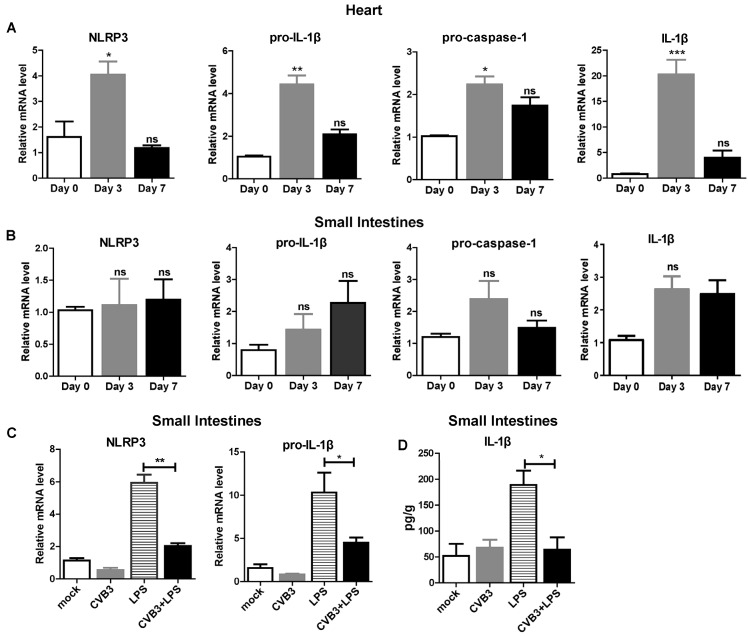
CVB3 infection inhibits NLRP3 inflammasome activation and IL-1β production in the small intestine. (**A**,**B**) Male BALB/c mice were intraperitoneally injected with a 1000 PFU dose of CVB3. On day 3 p.i., the hearts (**A**) and the small intestines (**B**) were harvested for the extraction of RNA. The mRNA levels of NLRP3, pro-caspase-1, pro-IL-1β, and mature IL-1β were measured via RT-PCR. (**C**) Male BALB/c mice were intraperitoneally injected with a 1000 PFU dose of CVB3. Three days later, mice were injected with 10 mg/kg of LPS. After 6 h, the mRNA levels of NLRP3 and pro-IL-1β in the small intestines were assessed via RT-PCR. (**D**) IL-1β levels of small intestine homogenate was measured via ELISA. Data are representative as means (SD) of at least three independent experiments. Values are represented as means ± SD. * *p <* 0.05; ** *p <* 0.01; *** *p <* 0.001.

CVB3 is transmitted via the fecal–oral route. As an enterovirus, CVB3 first enters the intestine epithelium before infecting the pancreas and heart, resulting in significant myocarditis and pancreatitis, but only mild intestinal inflammation [14]. In this study, we aimed to investigate whether the NLRP3 inflammasome senses CVB3 invasion and whether CVB3 infection stimulates the activation of the NLRP3 inflammasome. To address this, we established a CVB3-infected mouse model via intraperitoneal injection (i.p.) of 1000 PFU of CVB3 into BALB/c mice, and examined the mRNA levels of NLRP3, pro-caspase-1, and IL-1β in the heart and the small intestine tissues using quantitative PCR. Consistent with previous reports [8], CVB3 infection significantly induced the mRNA expression of NLRP3, pro-IL-1β, pro-caspase-1, and mature IL-1β in the heart tissues (Figure 1A). However, CVB3 infection did not induce the expression of NLRP3, pro-IL-1β, pro-caspase-1, or mature IL-1β in the small intestine (Figure 1B). The intestine is one of the organs with high exposure to external pathogens. Thus, we next explored the regulatory effect of CVB3 infection on LPS-induced NLRP3 inflammation in the small intestine. On day 3 after infection, CVB3-infected mice were treated with 10 mg/kg LPS (i.p.). As shown in Figure 1C,D, CVB3 infection alone did not activate the NLRP3 inflammasome, while LPS-stimulated mice showed significantly upregulated NLPR3. However, less NLRP3 and pro-IL-1β in the small intestine was observed in CVB3-infected mice with LPS stimulation than in control mice treated with LPS alone (Figure 1C). Moreover, secreted IL-1β in the small intestine was also strongly decreased in CVB3-infected mice after LPS stimulation compared with the LPS-treated control mice (Figure 1D). These results suggest that CVB3 infection could significantly inhibit NLRP3 expression and IL-1β secretion in the small intestine.

### 3.2. CVB3 Inhibits NLRP3 Inflammasome Activation and IL-1β Secretion in Macrophages

**Figure 2:**
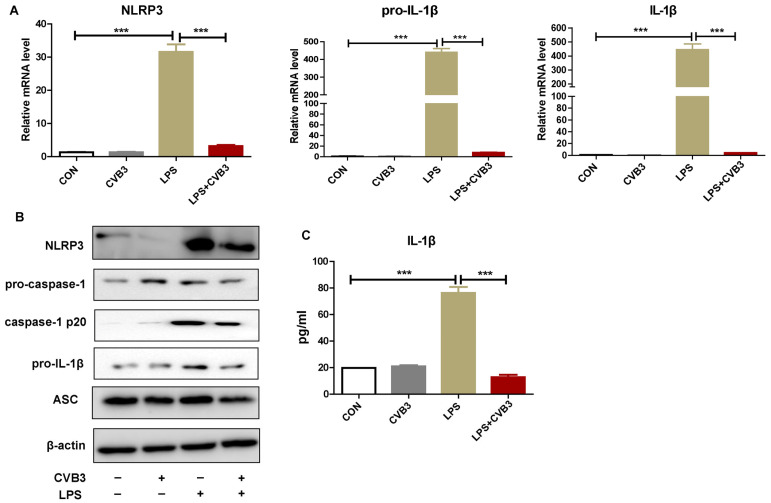
CVB3 inhibits NLRP3 inflammasome activation and IL-1β secretion in macrophages. Primary peritoneal macrophages were stimulated with 1 μg/mL of LPS for 6 h with or without pretreatment with CVB3 (MOI = 5) overnight. (**A**) The cells were collected for the extraction of RNA, and the mRNA levels of NLRP3, pro-IL-1β, and mature IL-1β were measured via RT-PCR. (**B**) The cells were lysed and the protein levels of NLRP3, pro-caspase-1, caspase p20, pro-IL-1β, and ASC were detected via Western blotting. (**C**) The small intestinal homogenate was collected and the protein level of IL-1β was analyzed via ELISA. Data are presented as means (SD) of at least three independent experiments. Values are presented as means ± SD. *** *p <* 0.001.

IL-1β is mainly produced by macrophages that express high levels of NLRs, including NLRP3 [11]. Thus, in this study, peritoneal macrophages were isolated and infected with CVB3, followed by a challenge with LPS. Then, NLRP3 inflammasome activation and IL-1β secretion were evaluated. The results show that in peritoneal macrophages, CVB3 alone did not induce NLRP3 inflammasome activation or IL-1β secretion, while LPS stimulation alone induced higher levels of NLRP3, pro-IL-1β, and mature IL-1β. However, in CVB3-infected peritoneal macrophages treated with LPS, NLRP3 inflammasome activation and IL-1β production were notably attenuated. The levels of NLRP3, pro-IL-1β, mature IL-1β (Figure 2A), pro-caspase-1, caspase p20, ASC (Figure 2B), and IL-1β production in the supernatants (Figure 2C) of CVB3-infected peritoneal macrophages were obviously decreased upon LPS stimulation compared with those without CVB3 infection. Collectively, these results indicate that CVB3 infection may alleviate NLRP3 inflammasome activation by suppressing caspase-1 activation and IL-1β maturation.

### 3.3. CVB3 Inhibits LPS-Trigged NF-κB Signaling Pathway

**Figure 3:**
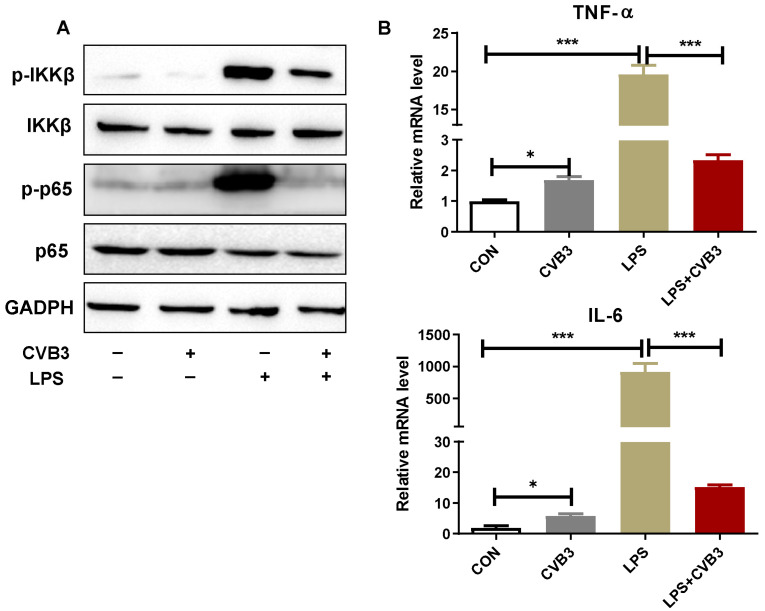
CVB3 inhibits LPS-activated NF-κB signaling pathway. Primary peritoneal macrophages were stimulated with 1 μg/mL of LPS for 6 h with or without pretreatment with CVB3 (MOI = 5) overnight. (**A**) The expression of the phosphorylation of p65 and IKKβ, total p65, and total IKKβ was detected via Western blotting. (**B**) The mRNA of IL-6 and TNF-α in cells was analyzed via RT-PCR. Data are presented as means (SD) of three independent experiments. Values are presented as means ± SD. * *p <* 0.05; *** *p* < 0.001.

The priming signal of NLRP3 inflammasome activation eventually results in the activation of the transcription factor NF-κB and the subsequent upregulation of NLRP3 and pro-IL-1β. Therefore, we first tested whether CVB3 impairs LPS-induced NF-κB activation. The result of Western blotting showed that CVB3 alone hardly induced IKKβ and p65 phosphorylation in peritoneal macrophages, whereas LPS alone stimulated high levels of their phosphorylation. However, in CVB3-infected peritoneal macrophages, the LPS-induced phosphorylation of IKKβ and p65 was significantly attenuated (Figure 3A). As expected, the IL-6 and TNF-α secretion induced by LPS were also significantly inhibited by CVB3 pre-infection (Figure 3B). Taken together, CVB3 could significantly inhibit LPS-stimulated activation of the NF-κB pathway, which implies that CVB3 may inhibit NLRP3 inflammasome activation by suppressing the NF-κB signaling pathway.

### 3.4. CVB3 Inhibits NLRP3 Inflammasome Activation by Decreasing ROS Production

**Figure 4:**
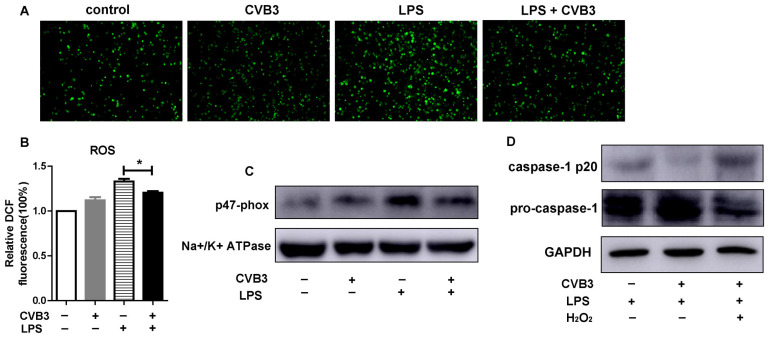
CVB3 suppresses NLRP3 inflammasome activation by inhibiting ROS production. (**A**,**B**) Peritoneal macrophages were pretreated with CVB3 (MOI = 5) overnight, and then, stimulated with 1 μg/mL of LPS. After 1 h, DCF fluorescence was detected using a fluorescence microscope (**A**) or analyzed using a microplate reader (**B**). (**C**) The membranes were prepared and the levels of p47-phox and an Na^+^/K^+^ ATPase were analyzed via Western blotting. (**D**) CVB3 pretreated peritoneal macrophages were stimulated with 5 mM H_2_O_2_ and LPS. After 1 h, pro-caspase-1 and cleaved caspase-1 were measured via Western blotting. Data are presented as means (SD) of at least three independent experiments. Values are presented as means ± SD. * *p* < 0.05.

The second signal of NLRP3 inflammasome activation is delivered from potassium efflux, calcium influx, ROS production, or lysosomal rupture. To investigate whether CVB3 affects the second signal for NLRP3 inflammasome activation, CVB3-infected peritoneal macrophages were stimulated with LPS. We found that LPS-induced intracellular ROS production was reduced in CVB3-infected peritoneal macrophages (Figure 4A,B). NADPH oxidase, a multi-subunit enzyme that donates an electron from nicotinamide adenine dinucleotide phosphate (NADPH) to oxygen, is thought to be the source of ROS production. NADPH oxidase consists of membrane-bound factors and cytosolic factors, including p47-phox, which is regarded as the NADPH oxidase organizer, and the membrane p47-phox level indicates the activation of NADPH oxidase [15]. Thus, we investigated whether CVB3 affects p47-phox upregulation induced by LPS. As shown in Figure 4C, membrane p47-phox was expressed at low levels in resting macrophages, and after CVB3 infection, its expression was only mildly upregulated. However, LPS induced p47-phox expression in peritoneal macrophages, and this upregulation was potently inhibited by CVB3 infection (Figure 4C). To further confirm this, we added exogenous H_2_O_2_ to macrophages, which effectively eliminated the inhibitory effect of CVB3 on the activation of the NLRP3 inflammasome (Figure 4D). Therefore, these results imply that CVB3 inhibits NLRP3 inflammasome activation by suppressing p47-phox upregulation, NADPH oxidase activation, and ROS generation, thereby impairing the second signal of NLRP3 inflammasome activation.

### 3.5. CVB3 Increases the Susceptibility of Mice to E. coli Infection

**Figure 5:**
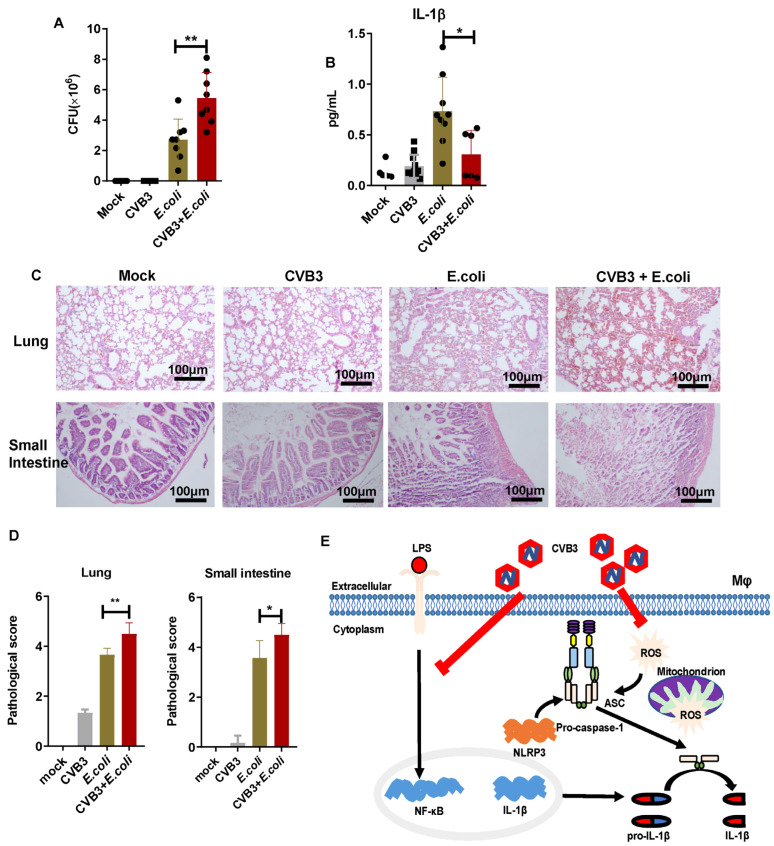
CVB3 infection increases the susceptibility of mice to *E. coli* ATCC25922 infection. Male BALB/c mice were intraperitoneally injected with a 1000 PFU dose of CVB3. On day 3 p.i., mice were infected i.p. with 2.0 × 10^7^ colony-forming units of *E. coli* for 18 h. (**A**) The bacterial burdens of control mice and CVB3-infected mice in the peritoneal lavage fluid were counted. (**B**) The IL-1β level in the peritoneal lavage fluid was detected via ELISA. (**C**) H&E staining and (**D**) pathological scoring of small intestines and lungs were performed to evaluate the tissue injury. Scale bar: 100 μm. (**E**) Postulated mechanism of the inhibitory effect of CVB3 infection on NLRP3 activation. CVB3 inhibits NLRP3 inflammasome activation and IL-1β production by suppressing NF-κB pathway and ROS production in macrophages. Data are presented as means (SD) of at least three independent experiments. Values are presented as means ± SD. * *p* < 0.05, ** *p* < 0.01.

The Gram-negative intracellular bacteria *E. coli* can trigger NLRP3 inflammasome activation and induce IL-1β release. The activated NLRP3 pathway can then inhibit bacterial replication [16]. Therefore, we investigated whether CVB3 infection increases susceptibility to *E.* in Figure 5C, increased *coli* infection by attenuating NLRP3 inflammasome activity and IL-1β production. First, male BALB/c mice were infected with 1000 PFU CVB3 (i.p.), and then, after 3 days, these mice were infected with *E. coli* (ATCC25922) for another 18 h (i.p.). Compared to mice infected with *E. coli* alone, mice infected with CVB3 following *E. coli* infection showed significantly higher bacterial counts but much lower IL-1β levels in the peritoneal lavage fluid (Figure 5A,B). As shown injury and inflammatory cell infiltration of the lung were observed in CVB3- and *E. coli*-infected mice compared with mice infected with *E. coli* only. Consistent with this, tissue necrosis and inflammation in the small intestines were observed after infection with *E. coli*, and pretreatment with CVB3 could significantly aggravate the inflammatory infiltration. Collectively, our data implicate that CVB3 infection could increase the susceptibility of mice to *E. coli* infection, possibly by inhibiting the activation of the NLRP3 inflammasome and the production of IL-1β.

## 4. Discussions

A large number of studies have shown that the activation of the NLRP3 inflammasome is essential for the host immune response against infections. On the one hand, if NLRP3 inflammasome activation is blocked, the immune response against pathogenic microorganisms cannot be effectively induced, resulting in severe infections. On the other hand, overwhelming activation of the NLRP3 inflammasome and the excessive production of inflammatory cytokines would lead to excessive cell death and severe pathological damage [17]. However, the majority of studies have shown that the activation of the NLRP3 inflammasome is more favorable for protective anti-infective immunity and pathogen clearance.

There is a complex relationship between viral infection and NLRP3 inflammasome activation. Viruses can be recognized by NLRP3 molecules in host cells, which then induce the assembly of inflammasome complexes and trigger the activation of downstream inflammatory signaling pathways to inhibit viral replication in host cells [18,19]. Meanwhile, viruses can also express non-structural proteins to impair NLRP3 inflammasome assembly and activation, thus escaping the host immune response. Recent studies have shown that enterovirus infection can activate the NLRP3 inflammasome [8], and that enterovirus 2B and 3D proteins can trigger NLRP3 inflammasome activation during EV71 and CVB3 infection [9,10]. In 2017, Tschope et al. reported that mice with defective cytoplasmatic pattern recognition receptors and nucleotide-binding oligomerization domain 2 (NOD2) had decreased CVB3-induced VMC [18]. In 2019, Chen et al. also reported that NLRP3 deficiency exacerbated enterovirus infection in mice, suggesting that NLRP3 inflammasome activation may facilitate the inhibition of enterovirus infection [19]. However, another study reported that inhibiting caspase-1 activation after CVB3 infection alleviated the symptoms of VMC.

Most studies at the cellular level suggest that CVB3 infection can directly activate the NLRP3 inflammasome pathway in cardiomyocytes [8]. However, CVB3 viral proteins can also directly degrade NLRP3’s components, thereby inhibiting its activation [9]. Therefore, the role and mechanism of the NLRP3 inflammasome in CVB3 viral infection are complex and need to be further explored. Macrophages are the main producers of IL-1β [11]. However, currently, there is only one report demonstrating that the CVB3 capsid proteins VP1 and VP2 triggered NLRP3 inflammasome activation in macrophages and effectively promoted the production of IL-1β by macrophages, thereby exacerbating VMC [20]. Herein, we focused on the role of CVB3 in macrophages and found that CVB3 pre-infection in macrophages could significantly inhibit LPS-induced NLRP3 inflammasome activation and IL-1β production.

CVB3, which is transmitted via the fecal–oral route, replicates at a lower level in the small intestine, causing mild local inflammation; then, it spreads to the pancreas and heart through blood circulation, causing severe pancreatitis and myocarditis [14]. The small intestine is one of the organs most likely to be exposed to pathogenic microorganisms. The NLRP3 inflammasome has also been implicated in cellular responses to LPS [21]. Therefore, we first examined NLRP3 inflammasome activation upon LPS stimulation in the small intestines of CVB3-infected mice. We found that CVB3 pre-infection could significantly inhibit the production of IL-1β and the expression of NLRP3 in the small intestine. Considering that CVB3 can hardly replicate in the intestinal epithelial cells, we conducted further experiments in macrophages. Generally, the activation of the NLRP3 inflammasome requires two signals. The first signal is dependent on the activation of NF-κB, and the second signal is delivered from potassium efflux, calcium influx, ROS production, or lysosomal rupture. We found that CVB3 inhibits NLRP3 inflammasome activation and IL-1β secretion in macrophages by inhibiting the NF-κB signaling pathway and ROS production. Inflammasome activation is important for anti-bacterial immunity, and our data demonstrate that CVB3 infection could increase the susceptibility of mice to *E. coli* infection, possibly by inhibiting the activation of the NLRP3 inflammasome and the production of IL-1β by attenuating NLRP3 inflammasome activity.

Collectively, our results indicate that CVB3 infection inhibited NLRP3 inflammasome activation and IL-1β production in macrophages through the suppression of the NF-κB signaling pathway and ROS production (Figure 5E). The findings of this study shed light on a novel mechanism of CVB3 in suppressing NLRP3 inflammasome activation, and may contribute to the understanding of the relationship between viral infection and innate immune responses. Additionally, our findings may provide experimental evidence for clinical antiviral treatment and drug development for CVB3 infection.

## Figures and Tables

**Table 1:** Primers.

	Sense Strand (5′-3′)	Anti-Sense Strand (5′-3′)	Size (bp)
NLRP3	AGGAGGAAGAAGAAGAGAGGA	AGAGACCACGGCAGAAGC	132 bp
pro-IL-β	TCTTTGAAGTTGACGGACCC	TGAGTGATACTGCCTGCCTG	135 bp
IL-β	GAAATGCCACCTTTTGACAGTG	TGGATGCTCTCATCAGGACAG	116 bp
pro-caspase-1	GGGCCCCAGGCAAGCCAAATC	AGGGCAAGACGTGTACGAGTGGT	302 bp
TNF-α	CGGTGCCTATGTCTCAGCCT	GAGGGTCTGGGCCATAGAAC	150 bp
IL-6	AGTTGCCTTCTTGGGACTGA	TCCACGATTTCCCAGAGAAC	159 bp
GAPDH	TGGATTTGGACGCATTGGTC	TTTGCACTGGTACGTGTTGAT	211 bp

## Data Availability

The data presented in this study are available on request from the corresponding author.
